# Psychological distress and associated factors among Palestinian advanced cancer patients: A cross-sectional study

**DOI:** 10.3389/fpsyg.2022.1061327

**Published:** 2022-12-02

**Authors:** Hammoda Abu-Odah, Alex Molassiotis, Ivy Y. Zhao, Jing Jing Su, Matthew J. Allsop

**Affiliations:** ^1^School of Nursing, The Hong Kong Polytechnic University, Kowloon, Hong Kong SAR, China; ^2^Nursing and Health Sciences Department, University College of Applied Sciences (UCAS), Gaza, Palestine; ^3^College of Arts, Humanities and Education, University of Derby, Derby, United Kingdom; ^4^Academic Unit of Palliative Care, Leeds Institute of Health Sciences, University of Leeds, Leeds, United Kingdom

**Keywords:** advanced cancer, anxiety, depression, Gaza, psychological distress

## Abstract

**Objective:**

There is limited research exploring the experiences of people living with advanced cancer in the Gaza Strip (GS), Palestine. Thus, this study aimed to determine the level of psychological distress, anxiety, and depression among advanced cancer patients in the GS and identify factors associated with a high level of distress.

**Materials and methods:**

A secondary analysis was performed using primary data from a larger study focusing on supportive care needs in advanced cancer patients in GS. Three hundred sixty-one patients agreed to participate and filled out the Distress Thermometer (DT) and the Hospital Anxiety and Depression Scale (HADS). Multivariate logistic regression was conducted to identify factors associated with high distress levels.

**Results:**

Over two-thirds of advanced cancer patients (70.6%) reported a high level of distress. They also reported a significantly higher distress level than patients with early cancer (96.5 vs. 3.5%; *p* = 0.001). About 92.8% of participants reported depression and anxiety symptoms. Physical, emotional, and practical problems were the primary sources of distress. Breast cancer patients were more likely to have psychological distress than colon and stomach cancer patients. Newly diagnosed patients had a higher level of anxiety, depression, and distress than those who had a cancer diagnosis for an extended period.

**Conclusion:**

Patients with advanced cancer in the GS exhibited a significantly higher level of psychological distress, depression and anxiety than patients with advanced cancer elsewhere. Efforts should be made to identify psychological distress as a routine part of oncology practice. Future research should further explore the causes of psychological distress in cancer patients in conflict zones and feasible mitigation strategies.

## Introduction

A diagnosis of cancer often negatively affects patients’ physical, psychological, spiritual, and financial status and their social relationships (Boen et al., 2018). Psychological distress has been defined as “a multifactorial unpleasant emotional experience of a psychological, social and/or spiritual nature that may interfere with the ability to cope effectively with cancer, its physical symptoms, and its treatment” (Holland and Alici, 2010). Patients with advanced cancer report a considerably higher level of psychological distress due to their life-threatening diagnosis, treatment-related side effects, financial burden and intensified family and social relationships (Chou et al., 2022). Living in stressful social situations, like conflict zones, is also a risk factor for depression and other types of psychological distress, and both depression and stress appear to increase cancer mortality (Steel et al., 2009; Slavich and Irwin, 2014; Bethea et al., 2016; Bortolato et al., 2017).

Psychological distress is associated with poor treatment adherence (Yee et al., 2017) and physical symptoms, such as insomnia, pain, fatigue and anorexia, which negatively impact the oncological treatment process (van Laarhoven et al., 2011). 20–40% of advanced cancer patients suffer from depression and anxiety (Diaz-Frutos et al., 2016). Furthermore, depression and hopelessness can undermine the quality of life and have a detrimental impact on survival (Batty et al., 2017).

The experiences of people living with advanced cancer in the Arab world have not been well studied (Hamadeh et al., 2017). One study from Saudi Arabia showed that psychological distress negatively influenced cancer patients’ quality of life, treatment compliance and duration of hospitalizations (Almigbal et al., 2019). In Jordan, Naser et al. (2021) reported a high level of depression and anxiety among cancer patients (23.4% and 19.1–19.9%, respectively), while in Lebanon, the prevalence of major depression among 102 breast cancer patients reached 30.1% (Abou Kassm et al., 2018). But these studies were limited either by focusing only on depressive and anxious symptoms (Abou Kassm et al., 2018; Naser et al., 2021) or by small sample sizes or specific cancer types (Abou Kassm et al., 2018). Further, because of the large variations across countries in wealth, health expenditures, quality of services, availability of treatment and palliative care, these results are not generalizable. There are few data on the experience of living with advanced cancer in lower-income settings and conflict zones such as the GS.

The health system in the GS is fragmented and lacks systematic policies and governance (AlKhaldi et al., 2020). Healthcare services, such as palliative care and advanced cancer treatments, are limited or unavailable (World Health Organization, 2019; Abu et al., 2020; Abu-Odah et al., 2022a). Financial resources are scarce, poverty levels are high, financial and administrative coordination are poor (Abu Hamad et al., 2016). The political conflict between the ministries of health in Gaza and the West Bank negatively impacts the development and enhancement of healthcare services provided to cancer patients. Furthermore, the ongoing conflict with Israel in the GS has heightened patients’ stress and increased the chance of death due to a lack of medications, medical equipment and underdeveloped transfer procedures (ElMokhallalati et al., 2022; Abu-Odah et al., 2022b).

A few studies have explored the quality of life of patients living with cancer in the GS (Nasser Ibrahim Abu El and MysoonKhalil Abu El, 2015; Salah et al., 2018; Shamallakh and Imam, 2017), and two focus on psychological factors associated with cancer. Bseiso and Thabet (2017) examined the relationship between siege stressors, depression, and anxiety among cancer patients, and ElMokhallalati et al. (2022) examined cancer patients’ quality of life and symptom burden. Neither study explored the level of psychological distress, anxiety, and depression among people living with advanced cancer in GS. Therefore, this study seeks to determine the level of psychological distress, anxiety, and depression among advanced cancer patients in the GS.

## Materials and methods

### Study design

A secondary analysis was performed using primary data from a larger study focusing on supportive care needs in advanced cancer patients in GS. The parent study employed a multi-method research design to comprehensively explore the factors and needs associated with the integration of a PC program in the Palestinian healthcare system from different key stakeholders (patients with cancer, healthcare professionals, and policymakers; Abu-Odah, 2022b). Following completion of the primary study, we were keen to utilize the rich dataset generated, including the opportunity to undertake an analysis focused specifically on psychological distress. Secondary data analysis is a valuable method for supporting exploration of supplementary research questions (Johnston, 2017).

### Setting

Participants were recruited from the oncology departments of two main governmental hospitals in the GS (European Gaza Hospital and Al-Shifa Hospital; Palestinian Ministry of Health, 2022).

### Characteristics of the participants and calculation of sample size

Participants were recruited through a convenience sampling method. The eligibility criteria were: (i) being diagnosed with stage III or IV cancer according to their medical records; (ii) being 18 years of age or above; (iii) being treated at one of the above-mentioned hospitals used as recruitment sites; (iv) visited the cancer centers’ out-patient department for follow-up treatment, and (v) being physically able to complete the survey for the study. Patients with cognitive impairment (e.g., caused by brain tumors) were excluded.

The sample size calculation followed the Thompson formula (Thompson, 2012, pp. 59–60). There were 8,903 cancer patients registered in the GS; however, no data were available about the number of patients with advanced stages. Assuming around half of all patients were living with advanced disease, the estimated sample size was 355 patients. The target sample was increased to 380 participants to account for an anticipated non-response rate of 7.1%, as informed from an earlier Palestinian study (ElMokhallalati et al., 2022).

### Study procedure

The research team approached the information technology department of the two recruiting hospitals and obtained a list of patients’ names who had made appointments for treatment. A patient list was forwarded to the oncology department to exclude non-eligible patients. A registered oncology nurse approached potential participants and introduced the study aim, significance, consent procedures, participant rights of withdrawal and details of participation. Patients were also provided with a detailed study information sheet alongside the questionnaire pack. Patients were informed of the voluntary nature of participation and their right to withdraw at any time without prejudice. All participants signed a consent form to provide informed consent prior to participation.

### Assessment scales

#### Sociodemographic and clinical data

A study-specific collection tool was developed for recording demographic and clinical information for each participant, including age, sex, marital status, education, employment, living conditions, and source of income. Clinical data included diagnosis, duration since diagnosis, clinical stage at initial diagnosis, and current and completed treatments (surgery, chemotherapy, radiotherapy, and others).

#### Distress thermometer scale

The Arabic version of the Distress Thermometer (DT) scale was used to identify levels of distress (Alosaimi et al., 2018). No modifications were applied to the original Arabic DT, as it adhered to and matched the spoken language of Palestinians. DT is a one-item, 11-point visual scale ranging from 0 (no distress) to 10 (high distress), covering 36 problems categorized into five domains: physical, emotional, practical, family, and spiritual concerns. The DT scores were divided into two categories: <6 = non-distressed patients and ≥6 = distressed patients. A DT cut-off score of 6 or above is the appropriate point to be utilised for identifying patients with a high level of distress (Goebel and Mehdorn, 2011; Renovanz et al., 2013). The DT scores were divided into two categories: <6 = non-distressed patients and ≥6 = distressed patients. The DT has been translated and validated into Arabic (Alosaimi et al., 2018).

#### The hospital anxiety and depression scale

The Hospital Anxiety and Depression Scale (HADS) was used to measure participants’ anxiety and depression levels (Terkawi et al., 2017). It is comprised of 14 items assessing two domains: anxiety and depression. The scores in each subscale are calculated and determined to fall under one of the two categories: normal cases (0–7), borderline cases (8–10), and cases (11–21; Zigmond and Snaith, 1983). HADS total score ≥15 indicated that patients had anxiety and depression signs. The translated and validated Arabic version of the HADS was used (Terkawi et al., 2017).

### Statistical methods

The Statistical Package for the Social Sciences (SPSS) software version 25 was used for data analysis. Descriptive statistics were used to summarise the participants’ sociodemographic characteristics, clinical data, and psychological well-being. Graphical (histogram) and numerical tests (Kolmogorov–Smirnov test and the Shapiro–Wilk test) were used to assess the normal distribution of the data. A chi-squared test (*x*^2^) and independent samples t-test were conducted as appropriate to identify variables associated with a high level of distress. All variables with a value of *p* ≤ 0.25 in *x*^2^ and *t*-test were selected for multivariate logistic regression. A value of 0.25 was based on previous literature (Bendel and Afifi, 1977; Mickey and Greenland, 1989). All statistical analyses were two-tailed, and a *p* < 0.05 was considered significant.

### Ethical considerations

Ethics permission was obtained from the Ethical Review Committee of The Hong Kong Polytechnic University (HSEARS20200414006) from where this study was led. The study was also approved by the Palestinian Ministry of Health in the GS (476303) and the study hospitals. Written informed consent was obtained before data collection.

## Results

### Characteristics of participants

Of the 404 advanced cancer patients invited to participate in the study, 361 patients did so. Just over half (52.1%) of the participants were male. Participants had a mean age of 50.02 ± 14.9. Around two-thirds of participants (68.8%) had a monthly income of less than 250 USD. For clinical characteristics, most patients were diagnosed with breast (21.3%), colon (15.5%), and lung (9.4%) cancers. 80.9% of participants were undergoing chemotherapy treatment. The detailed sociodemographic and clinical characteristics of study participants are presented in Table 1.

**Table 1:** Sociodemographic and clinical characteristics of participants (*N* = 361).

Participants’ characteristics	Total *N* = 361 (%)
Gender	
Male	188 (52.1)
Female	173 (47.9)
Marital status	
Single	36 (10.0)
Married	302 (83.7)
Separated	6 (1.7)
Widowed	17 (4.7)
Education	
Primary and less	48 (13.3)
Secondary	88 (24.4)
Tertiary	143 (39.6)
University	82 (22.7)
Working status	
None	169 (46.8)
Employee	101 (28.0)
Homemaker	91 (25.2)
Monthly Income* (USD; *N* = 346)	
Less than 250 USD	237 (68.5)
250–500	54 (15.6)
501–750	35 (10.1)
More than 750 USD	20 (5.8)
Diagnosis/type	
Breast	77 (21.3)
Colon	56 (15.5)
Lung	34 (9.4)
Bone	26 (7.2)
Prostate	16 (4.4)
Bladder	12 (3.3)
Thyroid	27 (7.5)
Lymphoid	23 (6.4)
Brain and neck	25 (6.9)
Stomach	17 (4.7)
Other	48 (13.3)
Clinical stage at initial diagnosis	
III	177 (49.0)
IV	184 (51.0)
Duration since diagnosis	
Within the last month (Newly diagnosed patients)	23 (6.4)
1–12 months ago,	128 (35.5)
Over 1 year^−3^ years ago	126 (34.9)
Over 3 years ago	84 (23.3)
Current treatment	
Chemotherapy	292 (80.9)
Radiation	26 (7.2)
Surgical	16 (4.4)
Other	27 (7.5)
Age (years; mean ± SD)	50.02 ± 14.9
DT (mean ± SD)	6.71 ± 2.47
HADS-Total (mean ± SD)	22.47 ± 5.55
HADS-D	11.14 ± 3.07
HADS-A	11.34 ± 3.37

DT = distress thermometer; HADS-A = hospital anxiety and depression scale-anxiety; HADS-D = hospital anxiety and depression scale-depression; HADS-T = hospital anxiety and depression scale-total; SD = standard deviation; USD = United States Dollar.

* Income is categorized according to the Palestinian labor laws.

### Frequency of distress

The mean DT score was 6.71 ± 2.47, ranging from 0 to10. 70.6% (*n* = 255) of advanced cancer patients reported a high level of distress (cutoff DT ≥ 6). 14.7% (*n* = 35) of patients reported extreme distress (distress at a level of 10). Findings also showed that advanced cancer patients reported significantly higher distress level than patients with early cancer (96.5 vs. 3.5%; *p* = 0.001).

**Figure 1:**
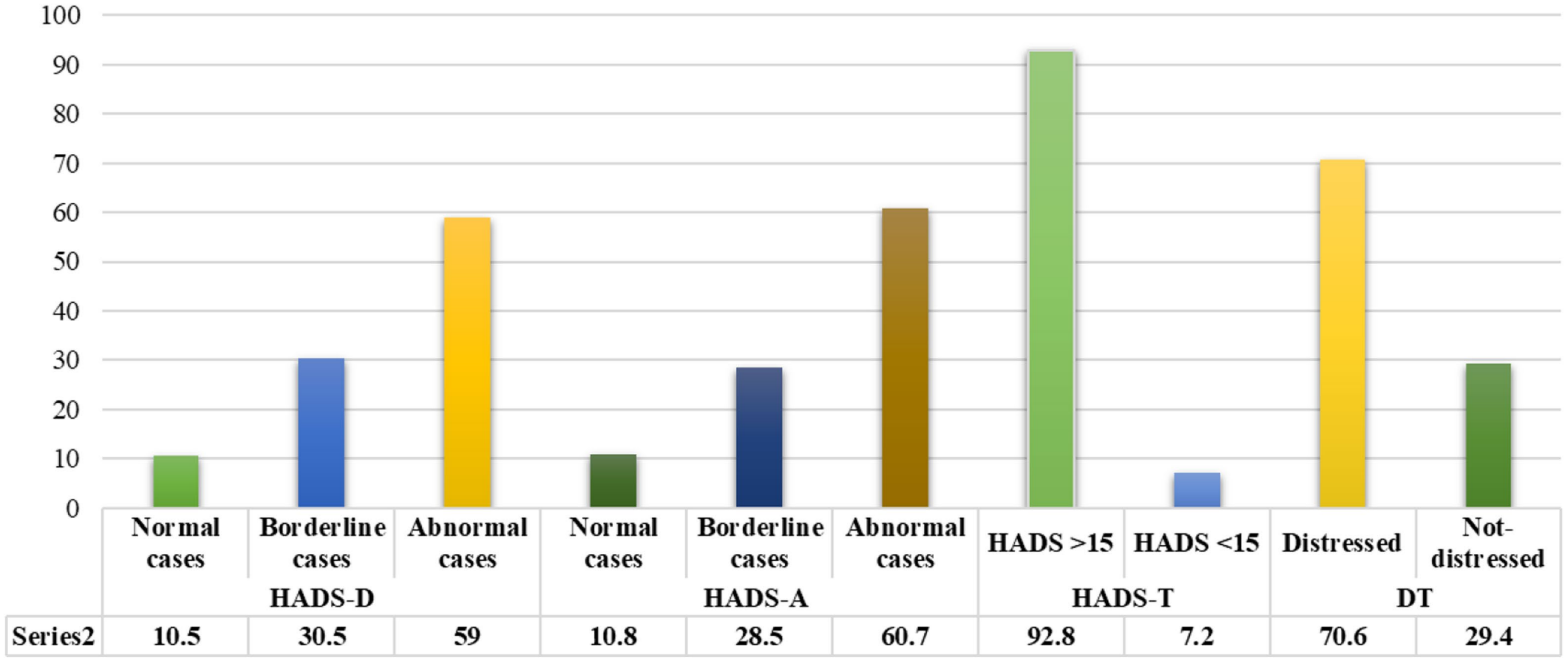
Frequency of distress among Palestinian advanced cancer patients (*N* = 361).

For the HADS-T, the mean score was 22.49 ± 5.55, ranging from 3 to 42, with 92.8% (*n* = 335) of participants reporting signs of anxiety and depression. The mean score of HADS-D was 11.14 ± 3.07 and was 11.34 ± 3.37 for HADS-A. More than half (*n* = 213, 59.0%) of participants were classified as clinical cases and 110 (30.5%) as borderline cases of depression. Two hundred nineteen participants (60.7%) had scores suggestive of anxiety cases, and 103 (28.5%) were borderline cases (Figure 1).

### Source of distress

**Figure 2:**
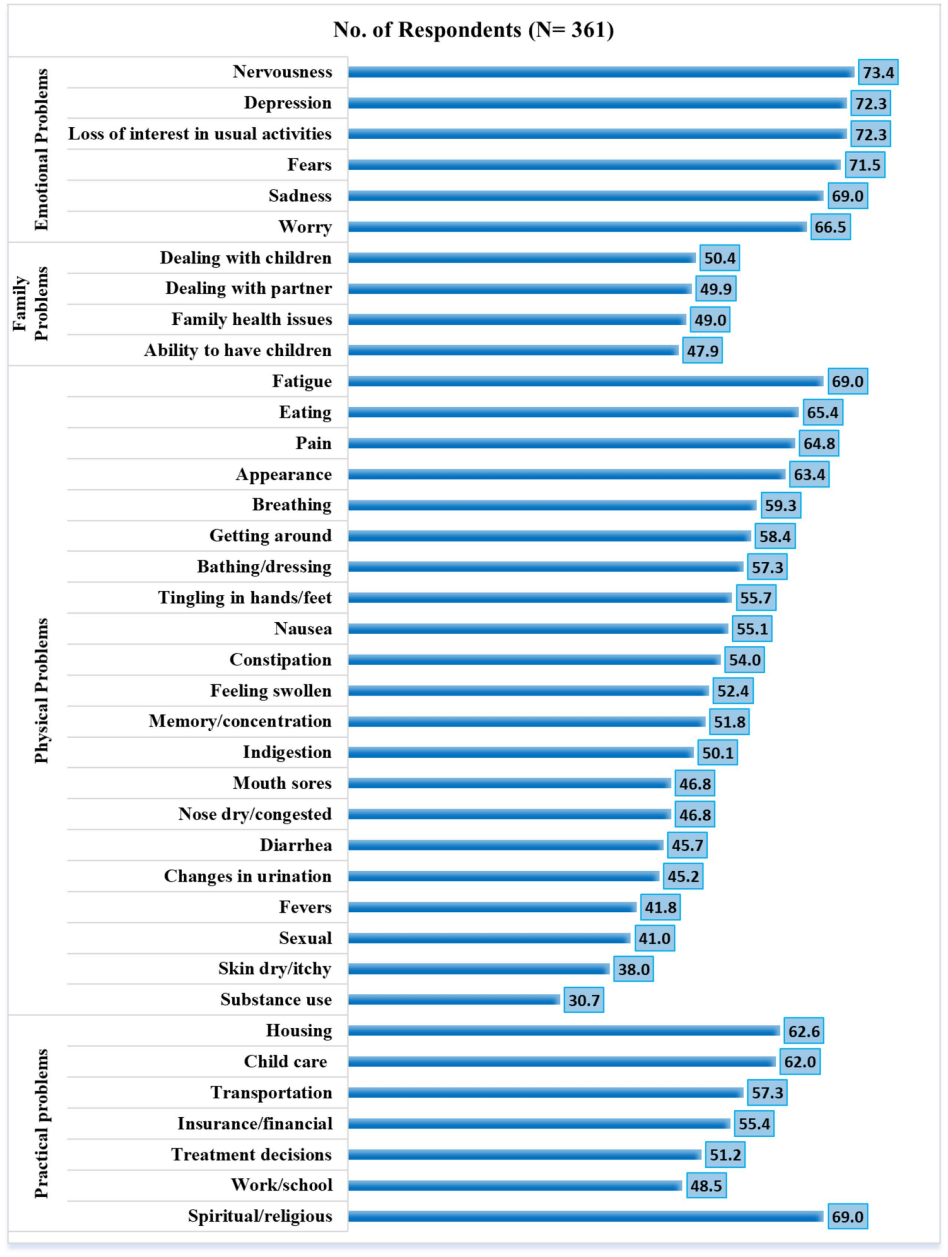
Percentage distribution of participants with respect to distress thermometer scale parameters.

The major sources of distress were related to physical (*n* = 355, 98.3%), emotional (*n* = 341, 94.5%), and practical problems (*n* = 308, 85.3%). Concerning DT problem lists, *x*^2^ showed that patients with a high distress level had more problems with insurance/financial aspects than those patients without distress (73.3 vs. 26.3%, *p* = 0.042) in the practical domain. Feeling swollen (75.5 vs. 24.3%, *p* = 0.028), getting around (76.3 vs. 23.7%, *p* = 0.005), indigestion (76.2 vs.23.0%, *p* = 0.019), memory/concentration (77.0 vs. 23.0%, *p* = 0.006), mouth sores (75.7 vs. 24.3%, *p* = 0.046), nausea (76.4 vs.23.6%, *p* = 0.008), nose dry/congested (75.5 vs. 24.3%, *p* = 0.046), pain (74.4 vs. 25.6%, *p* = 0.035), and sexual (78.4 vs. 21.6%, *p* = 0.007) were more physical frequent problems. A detailed frequency of problems list items and their association with distress levels presented in Table 2 and Figure 2.

**Table 2:** List of problems influencing distress levels.

DT problem Lists	Distress thermometer (DT)		*p*-value
	Distressed patients *N* = 255 (%)	Not-distressed patients *N* = 106 (%)	
Practical problems			
Childcare	165 (73.3)	59 (26.3)	0.107
Housing	163 (72.1)	63 (27.9)	0.422
Insurance/financial	150 (75.0)	50 (25.0)	**0.042**
Transportation	149 (72.0)	58 (28.0)	0.516
Work/school	126 (72.0)	49 (28.0)	0.581
Treatment decisions	132 (71.4)	53 (28.6)	0.760
Family Problems			
Dealing with children	137 (75.3)	45 (24.7)	0.051
Dealing with partner	128 (71.1)	52 (28.9)	0.791
Ability to have children	121 (69.9)	52 (30.1)	0.781
Family health issues	120 (67.8)	57 (32.2)	0.245
Emotional Problems			
Depression	189 (72.4)	72 (27.6)	0.231
Fears	189 (73.3)	69 (26.7)	0.084
Nervousness	191 (72.1)	74 (27.9)	0.319
Sadness	181 (72.2)	68 (27.3)	0.201
Worry	175 (72.9)	65 (27.1)	0.180
Loss of interest in usual activities	184 (70.5)	77 (29.5)	0.925
Spiritual/religious	174 (69.9)	75 (30.1)	0.637
Physical Problems			
Appearance	169 (73.8)	60 (26.2)	0.082
Bathing/dressing	151 (72.9)	56 (27.1)	0.264
Breathing	158 (73.8)	56 (26.2)	0.108
Changes in urination	114 (69.9)	49 (30.1)	0.791
Constipation	139 (71.3)	56 (28.7)	0.771
Diarrhea	116 (70.3)	49 (29.7)	0.898
Eating	169 (71.6)	67 (38.4)	0.577
Fatigue	176 (70.7)	73 (29.3)	0.977
Feeling swollen	143 (75.7)	46 (24.3)	**0.028**
Fevers	112 (74.2)	39 (25.8)	0.211
Getting around	161 (76.3)	50 (23.7)	**0.005**
Indigestion	138 (76.2)	43 (23.0)	**0.019**
Memory/concentration	144 (77.0)	43 (23.0)	**0.006**
Mouth sores	128 (75.7)	41 (24.3)	**0.046**
Nausea	152 (76.4)	47 (23.6)	**0.008**
Nose dry/congested	128 (75.5)	41 (24.3)	**0.046**
Pain	174 (74.4)	60 (25.6)	**0.035**
Sexual	116 (78.4)	32 (21.6)	**0.007**
Skin dry/itchy	112 (81.8)	25 (18.2)	**0.000**
Substance use	89 (80.2)	22 (19.8)	**0.008**
Tingling in hands/feet	152 (75.6)	49 (24.4)	**0.020**

Bold value indicates significant level. DT = distress thermometer.

### Factors associated with distress

Findings underscored that breast cancer patients were more likely to have psychological distress than patients with colon (OR = 2.62, 95%, CI 1.11–6.16) or stomach cancer (OR = 4.81, 95% CI 1.22–18.94). However, they had similar psychological distress levels with other cancer diagnostic groups. Patients with breast cancer were also more likely to have a high level of anxiety and depression than patients with colon (OR = 0.11, 95%, CI 0.02–0.71), bone (OR = 0.03, 95%, CI 0.00–0.40), lymphoma (OR = 0.08, 95%, CI 0.01–0.92), and stomach cancer (OR = 0.10, 95%, CI 0.00–0.13). Newly diagnosed patients were more likely to have high anxiety, depression, and distress than those diagnosed for longer. Physical problems independently influenced patients’ psychological distress (OR = 0.16, 95%, CI 0.04–0.60), anxiety and depression (OR = 4.29.8, 95%, CI 9.76–188.40; Table 3).

**Table 3:** Multivariate logistic regression model for both scales.

Variables	Distress thermometer scale (DT)					Hospital Anxiety and Depression Scale (HADS)				
	β	S.E.	Wald	OR (95% CI)	*p* –value	β	S.E.	Wald	OR (95% CI)	*p* –value
Monthly Income										
Less than 250 USD	Ref.	-	-	-	-	-	-	-	-	-
250–500	0.57	0.36	2.58	1.77 (0.88–3.57)	0.108	−1.16	0.62	3.48	0.31 (0.09–1.06)	0.062
501–750	0.44	0.47	0.85	1.54 (0.61–3.89)	0.356	2.26	1.31	2.97	9.57 (0.73–124.92)	0.085
More than 750 USD	−20.55	8599.95	0.00	0.00 (0.00–0.00)	0.998	−0.06	1.33	0.00	0.95 (0.07–12.81)	0.966
Diagnosis/Type										
Breast	Ref.	-	-	-	-	-	-	-	-	-
Colon	0.96	0.44	4.86	2.62 (1.11–6.16)	**0.027**	−2.20	0.94	5.42	0.11 (0.02–0.71)	**0.020**
Lung	−0.34	0.65	0.28	0.71 (0.20–2.53)	0.598	−1.58	1.37	1.32	0.21 (0.01–3.05)	0.251
Bone	0.18	0.61	0.09	1.20 (0.36–4.01)	0.766	−3.60	1.36	6.96	0.03 (0.00–0.40)	**0.008**
Prostate	−0.94	0.85	1.22	0.39 (0.07–2.06)	0.269	16.87	8504.22	0.00	2110.94 (0.00–0.00)	0.998
Bladder	0.97	0.70	1.96	2.65 (0.68–10.34)	0.162	16.57	10947.94	0.00	1571.42 (0.00–0.00)	0.999
Thyroid	0.32	0.54	0.34	1.37 (0.47–3.99)	0.561	−0.94	1.16	0.65	0.39 (0.04–3.82)	0.421
Lymphoid	1.14	0.61	3.49	3.12 (0.94–10.32)	0.062	−2.51	1.24	4.12	0.08 (0.01–0.92)	**0.042**
Brain and neck	−0.15	0.63	0.05	0.86 (0.25–2.97)	0.816	17.67	6586.33	0.00	4717.09 (0.00–0.00)	0.998
Stomach	1.57	0.70	5.04	4.81 (1.22–18.94)	**0.025**	−4.46	1.22	13.44	0.01 (0.00–0.13)	**0.000**
Other	0.87	0.47	3.40	2.40 (0.95–6.07)	0.065	−1.52	1.08	2.00	0.22 (0.03–1.80)	0.157
Clinical stage (IV) at initial diagnosis	0.02	0.29	0.01	1.02 (0.58–1.81)	0.941	−0.60	0.64	0.86	0.55 (0.16–1.95)	0.354
Duration since diagnosis										
Within the last month	Ref.	-	-	-	-	-	-	-	-	-
1–12 months ago,	−1.73	0.55	9.96	0.18 (0.06–0.52)	**0.002**	2.84	0.91	9.78	17.03 (2.88–100.66)	**0.002**
Over 1 year-3 years ago	−0.84	0.55	2.29	0.43 (0.15–1.28)	0.130	1.99	0.92	4.73	7.33 (1.22–44.15)	**0.030**
Over 3 years ago	−0.92	0.58	2.58	0.40 (0.13–1.23)	0.109	1.41	0.90	2.47	4.09 (0.71–23.61)	0.116
Practical problems	0.42	0.44	0.91	1.52 (0.64–3.60)	0.339	0.55	0.89	0.38	1.74 (0.30–10.05)	0.535
Emotional problems	−0.43	0.44	0.95	0.65 (0.27–1.54)	0.329	2.62	0.81	10.47	13.76 (2.81–67.34)	**0.001**
Physical problem	−1.86	0.69	7.21	0.16 (0.04–0.60)	**0.007**	6.06	1.93	9.87	429.84 (9.76–188.40)	**0.002**
Family problems						−1.18	0.80	2.17	0.31 (0.06–1.48)	0.141

HADS = hospital anxiety and depression scale; SD = standard deviation; USD = United States Dollar. Bold value indicates significant level.

## Discussion

This study showed high levels of psychological distress, anxiety and depression among patients living with advanced cancer in the GS. This is the first study to explore the psychological impact of advanced cancer in the region, presenting its magnitude and significant impact. Patients with breast cancer or a new cancer diagnosis were more likely to report higher levels of distress, alongside financial problems being common among participants reporting high levels of distress.

While psychological distress is common among cancer patients, the frequency of distress, anxiety and depression among the study population is higher than that reported in previous studies (Alosaimi et al., 2018; Guan et al., 2019; Abd El-Aziz et al., 2020; Veeraiah et al., 2022). For instance, in India, 60% of cancer patients reported moderate to high distress (Veeraiah et al., 2022). In China, 70.5% of the patients were distressed (Guan et al., 2019), and 46% of patients in Egypt had significant distress (Abd El-Aziz et al., 2020). There may be multiple drivers of the higher levels of psychological distress reported across this study population. These could include cancer treatment effects (O’Connor et al., 2019), but also may in part be influenced by the health system context. In the GS, there remains fragmentation across the healthcare system, shortages in diagnostic and treatment facilities for people living with cancer, alongside increased barriers to accessing timely and appropriate care due to travel limitations and exposure to conflict and violence (World Health Organisation, 2020). For those seeking to access cancer care outside Gaza, restrictions by Israeli or Egyptian authorities lead to few patients being able to leave Gaza and many patients dying while waiting for medical permits from authorities to cross borders (Palestinian Center for Human Rights, 2017). Future research is required to understand the causal factors of the elevated psychological distress determined in the study sample and the extent to which it is driven by repercussions of the ongoing socio-political context. Doing so will enable the development of appropriate interventional programs suited for delivery in Gaza, where there is currently very limited capacity across psychological services in cancer centres. Context-appropriate interventions may then form part of ongoing efforts to strengthen the capacity of healthcare services to deliver psychosocial and palliative care, evidence-based approaches to the management of distress for people with cancer, in the GS. For example, palliative care training has recently been integrated within the Islamic University of Gaza’s undergraduate medical curriculum, which includes a focus on psychosocial and spiritual aspects for pain and palliative care, alongside the introduction of a diploma course for postgraduate students (Coghlan et al., 2019). Furthermore, within confines of limited resources, food insecurity, and disrupted procurement of medicines and supplies, there continues to be excellence and resilience in the responses of health facilities, such as efforts during the COVID-19 pandemic to ensure continuity of care for people with cancer (Sabateen et al., 2022). However, irrespective of efforts to strengthen capacity of cancer and palliative care in the region, there remains a need for continued efforts to address ongoing political, economic and territorial restrictions that harm access to timely and appropriate for people with cancer.

The sources of psychological distress in our study are aligned with literature from the US, Jordan and Ireland (Ryan et al., 2012; Omran et al., 2017; McFarland et al., 2018). Fatigue, feeling swollen, pain, eating difficulties, worrying and feeling nervous, or loss of interest, were the main problem items significantly related to psychological distress in Gazan patients. A retrospective study in Mexico City has also reported that the psychological distress of patients with advanced cancer was significantly associated with transportation (Ascencio-Huertas et al., 2021). This was not found in this study, which may be related to the availability of support from non-governmental organizations covering transportation expenses for patients with cancer in Gaza, alongside the short distances travelling to hospitals in the GS.

Our study suggested that in the context of a healthcare system with universal health coverage, financial inequities are still a considerable concern related to psychological well-being. Existing evidence has highlighted cancer patients’ significant financial burden through the illness trajectory, contributing to greater distress, anxiety and depression, and symptom burden (Knaul et al., 2018; Malhotra et al., 2020). Since cancer patients’ major financial concern is the direct out-of-pocket expense for cancer treatment and care (e.g., nutritional supplements), universal health coverage was advocated to offset this burden on patients (Knaul et al., 2018). However, our findings suggest that universal health coverage is not the sole answer to addressing the financial inequities of Palestine for optimizing their psychosocial well-being. Due to the limited allocation of government funding to health expenditure and limited capacity of healthcare services, patients do not have access to the best available treatment under a universal health coverage scheme. For those with adequate financial resources, self-funding treatment from other countries is the only option available.

There were no associations reported between distress and demographic variables, with a new cancer diagnosis and having a diagnosis of breast cancer the only associated clinical variables. Earlier research similarly found no association between the DT and demographic variables (Herschbach et al., 2004; Holland et al., 2013), although other cancer diagnoses such as head and neck cancer have been associated with higher distress (Chiou et al., 2016). Our findings highlighted that levels of distress were high for participants around the time of diagnosis, which aligns with earlier work in, for example, Egypt (Alagizy et al., 2020). From the time of their diagnosis with cancer, patients may be concerned about the future and the spread of disease (Kwak et al., 2013). Psychological distress in cancer patients can extend along the cancer continuum; however, a cancer diagnosis remains a common source of distress (Cook et al., 2015). This study highlights the need to improve alignment with long-established recommendations of the US National Comprehensive Cancer Network (Ownby, 2019), for routine screening for distress in all cancer patients in the GS to facilitate its timely identification and management.

This study had several limitations. Our study instruments were not designed to assess social factors that may contribute to psychological distress such as exposure to discrimination or political violence, refugee status, or inability to travel. Also, the cross-sectional design of our study hindered us from determining the causation for any observed association. Adopting non-probability sampling methods made it difficult to generalize our findings to all patients. As 50% of the cancer patients in this study were at a late clinical stage, this may affect the study’s power, which is considered one of its limitations. More research is required that includes a variety of cancers types and phases (early vs. late). However, the two instruments enabled assessment of the distress, anxiety, and depression in Gazan advanced cancer patients.

## Conclusion

Gazan patients with advanced cancer exhibited a significantly higher level of psychological distress, depression and anxiety symptomatology than patients with advanced cancer elsewhere. Efforts should be made to improve routine screening and detection of distress in patients with advanced cancer to facilitate its timely identification and management. This will also require concurrent research to determine the causal factors leading to increased levels of psychological distress, depression and anxiety. This will provide requisite evidence needed to guide the development of interventional programs that can be offered where psychological needs are identified that are appropriate within the socio-political environment in which cancer care is being delivered in Gaza.

## Data availability statement

The raw data supporting the conclusions of this article will be made available by the authors, without undue reservation.

## Author contributions

HA-O conducted analyses and wrote the manuscript. HA-O and AM conducted data management. IZ and JS reviewed the manuscript. MA contributed to manuscript writing. All authors contributed to the article and approved the submitted version.

## Conflict of interest

The authors declare that the research was conducted in the absence of any commercial or financial relationships that could be construed as a potential conflict of interest.

## Publisher’s note

All claims expressed in this article are solely those of the authors and do not necessarily represent those of their affiliated organizations, or those of the publisher, the editors and the reviewers. Any product that may be evaluated in this article, or claim that may be made by its manufacturer, is not guaranteed or endorsed by the publisher.

## References

[ref1] Abd El-Aziz N. Khallaf S. Abozaid W. Elgohary G. Abd El-Fattah O. Alhawari M.. (2020). Is it the time to implement the routine use of distress thermometer among Egyptian patients with newly diagnosed cancer? BMC Cancer 20:1033. doi: 10.1186/s12885-020-07451-7, PMID: 33109093 PMC7592584

[ref2] Abou Kassm S. Hlais S. Khater C. Chehade I. Haddad R. Chahine J.. (2018). Depression and religiosity and their correlates in Lebanese breast cancer patients. Psycho-Oncology 27, 99–105. doi: 10.1002/pon.4386, PMID: 28125166

[ref3] Abu Hamad B. Skaik N. Abu-Odah H. (2016). Evaluation of palliative care services provided to cancer patients in the Gaza strip. J. US-Chin. Med. Sci. 13, 95–107. doi: 10.17265/1548-6648/2016.02.006

[ref4] Abu-Odah H. Mikati D. Arawi T. (2020). “Deconstructing palliative care in areas of armed conflict: needs, challenges, and concerns,” in Handbook of healthcare in the Arab world. ed. Laher I. (Switzerland: Springer International Publishing), 1–17.

[ref6] Abu-Odah H. Molassiotis A. Liu J. Y. W. (2022a). Assessment of the educational and health care system-related issues from physicians' and nurses' perspectives before developing a palliative care program within the Palestinian health care system: a cross-sectional study. J. Hosp. Palliat. Nurs. 24, E59–E75. doi: 10.1097/njh.0000000000000840 35085161

[ref7] Abu-Odah H. Molassiotis A. Liu J. Y. W. (2022b). Analysis of the unmet needs of Palestinian advanced cancer patients and their relationship to emotional distress: results from a cross-sectional study. BMC Palliat. Care 21:72. doi: 10.1186/s12904-022-00959-8, PMID: 35562732 PMC9106510

[ref8] Alagizy H. A. Soltan M. R. Soliman S. S. Hegazy N. N. Gohar S. F. (2020). Anxiety, depression and perceived stress among breast cancer patients: single institute experience. Middle East current. Psychiatry 27, 1–10. doi: 10.1186/s43045-020-00036-x

[ref9] AlKhaldi M. Kaloti R. Shella D. Al Basuoni A. Meghari H. (2020). Health system's response to the COVID-19 pandemic in conflict settings: policy reflections from Palestine. Glob. Public Health 15, 1244–1256. doi: 10.1080/17441692.2020.1781914, PMID: 32552389

[ref10] Almigbal T. H. Almutairi K. M. Fu J. B. Vinluan J. M. Alhelih E. Alonazi W. B.. (2019). Assessment of psychological distress among cancer patients undergoing radiotherapy in Saudi Arabia. Psychol. Res. Behav. Manag. 12, 691–700. doi: 10.2147/PRBM.S209896, PMID: 31693712 PMC6708396

[ref11] Alosaimi F. D. Abdel-Aziz N. Alsaleh K. AlSheikh R. AlSheikh R. Abdel-Warith A. (2018). Validity and feasibility of the Arabic version of distress thermometer for Saudi cancer patients. PLoS One 13, e0207364–e0207364. doi: 10.1371/journal.pone.0207364, PMID: 30427918 PMC6241127

[ref12] Ascencio-Huertas L. Allende-Pérez S. Pastrana T. (2021). Associated factors of distress in patients with advanced cancer: a retrospective study. Palliat. Suppor. Care 19, 447–456. doi: 10.1017/S1478951520001066, PMID: 33222720

[ref13] Batty G. D. Russ T. C. Stamatakis E. Kivimäki M. (2017). Psychological distress in relation to site specific cancer mortality: pooling of unpublished data from 16 prospective cohort studies. BMJ 356:j108. doi: 10.1136/bmj.j108, PMID: 28122812 PMC5266623

[ref14] Bendel R. B. Afifi A. A. (1977). Comparison of stopping rules in forward “stepwise” regression. J. Am. Stat. Assoc. 72, 46–53. doi: 10.1080/01621459.1977.10479905

[ref15] Bethea T. N. Palmer J. R. Rosenberg L. Cozier Y. C. (2016). Neighborhood socioeconomic status in relation to all-cause, cancer, and cardiovascular mortality in the black women's health study. Ethn. Dis. 26, 157–164. doi: 10.18865/ed.26.2.157, PMID: 27103765 PMC4836895

[ref16] Boen C. E. Barrow D. A. Bensen J. T. Farnan L. Gerstel A. Hendrix L. H.. (2018). Social relationships, inflammation, and cancer survival. Cancer Epidemiol. Biomark. Prev. 27, 541–549. doi: 10.1158/1055-9965.EPI-17-0836, PMID: 29475966 PMC5932225

[ref17] Bortolato B. Hyphantis T. N. Valpione S. Perini G. Maes M. Morris G.. (2017). Depression in cancer: the many biobehavioral pathways driving tumor progression. Cancer Treat. Rev. 52, 58–70. doi: 10.1016/j.ctrv.2016.11.004, PMID: 27894012

[ref18] Bseiso R. A. Thabet A. (2017). The relationship between siege stressors, anxiety, and depression among patients with cancer in Gaza strip. Health Sci. J. 11:499. doi: 10.21767/1791-809X.1000499

[ref19] Chiou Y. J. Chiu N. M. Wang L. J. Li S. H. Lee C. Y. Wu M. K.. (2016). Prevalence and related factors of psychological distress among cancer inpatients using routine distress thermometer and Chinese health questionnaire screening. Neuropsychiatr. Dis. Treat. 12, 2765–2773. doi: 10.2147/ndt.S118667, PMID: 27822049 PMC5087777

[ref20] Chou W. Y. S. Tiner J. Senft N. (2022). “Emerging challenges in advanced cancer care: opportunities for enhancing patient-centered communication,” in Psychological aspects of cancer. eds. Steel J. L. Carr B. I. (Switzerland: Springer).

[ref21] Coghlan R. Leng M. Shamieh O. Elessi K. Grant L. (2019). A role for palliative care in advancing health in conflict settings. Lancet 394:1324. doi: 10.1016/S0140-6736(19)31826-4, PMID: 31609226

[ref22] Cook S. A. Salmon P. Dunn G. Holcombe C. Cornford P. Fisher P. (2015). A prospective study of the association of metacognitive beliefs and processes with persistent emotional distress after diagnosis of cancer. Cogn. Ther. Res. 39, 51–60. doi: 10.1007/s10608-014-9640-x, PMID: 25657483 PMC4312385

[ref23] Diaz-Frutos D. Baca-Garcia E. García-Foncillas J. López-Castroman J. (2016). Predictors of psychological distress in advanced cancer patients under palliative treatments. Eur. J. Cancer Care 25, 608–615. doi: 10.1111/ecc.12521, PMID: 27271213

[ref24] ElMokhallalati Y. Alaloul E. Shatat M. Shneewra T. El Massri S. Shaer O.. (2022). The symptom burden and quality of life in cancer patients in the Gaza strip, Palestine: a cross-sectional study. PLoS One 17:e0262512. doi: 10.1371/journal.pone.0262512, PMID: 35025966 PMC8758072

[ref25] Goebel S. Mehdorn H. M. (2011). Measurement of psychological distress in patients with intracranial tumours: the NCCN distress thermometer. J. Neuro-Oncol. 104, 357–364. doi: 10.1007/s11060-010-0501-5, PMID: 21188470

[ref26] Guan B. Wang K. Shao Y. Cheng X. Hao J. Tian C.. (2019). The use of distress thermometer in advanced cancer inpatients with pain. Psycho-Oncology 28, 1004–1010. doi: 10.1002/pon.5032 30762263

[ref27] Hamadeh R. R. Borgan S. M. Sibai A. M. (2017). Cancer research in the Arab world: a review of publications from seven countries between 2000-2013. Sultan Qaboos Univ. Med. J. 17, e147–e154. doi: 10.18295/squmj.2016.17.02.003, PMID: 28690885 PMC5488814

[ref28] Herschbach P. Keller M. Knight L. Brandl T. Huber B. Henrich G.. (2004). Psychological problems of cancer patients: a cancer distress screening with a cancer-specific questionnaire. Br. J. Cancer 91, 504–511. doi: 10.1038/sj.bjc.6601986, PMID: 15238979 PMC2409853

[ref29] Holland J. C. Alici Y. (2010). Management of distress in cancer patients. J. Support. Oncol. 8, 4–12. 20235417

[ref30] Holland J. C. Andersen B. Breitbart W. S. Buchmann L. O. Compas B. Deshields T. L.. (2013). Distress management: clinical practice guidelines in oncology. J. Natl. Compr. Cancer Netw. 11, 190–209. doi: 10.6004/jnccn.2013.0027 23411386

[ref31] Johnston M. P. (2017). Secondary data analysis: a method of which the time has come. Qual. Quan. Methods Lib. 3, 619–626.

[ref32] Knaul F. M. Farmer P. E. Krakauer E. L. De Lima L. Bhadelia A. Jiang Kwete X.. (2018). Alleviating the access abyss in palliative care and pain relief—an imperative of universal health coverage: the lancet commission report. Lancet 391, 1391–1454. doi: 10.1016/S0140-6736, PMID: 29032993

[ref33] Kwak M. Zebrack B. J. Meeske K. A. Embry L. Aguilar C. Block R.. (2013). Trajectories of psychological distress in adolescent and young adult patients with cancer: a 1-year longitudinal study. J. Clin. Oncol. 31, 2160–2166. doi: 10.1200/JCO.2012.45.9222, PMID: 23650425

[ref34] Malhotra C. Harding R. Teo I. Ozdemir S. Koh G. C. H. Neo P.. (2020). Financial difficulties are associated with greater total pain and suffering among patients with advanced cancer: results from the COMPASS study. Support Care Cancer 28, 3781–3789. doi: 10.1007/s00520-019-05208-y, PMID: 31832824

[ref35] McFarland D. C. Shaffer K. M. Tiersten A. Holland J. (2018). Physical symptom burden and its association with distress, anxiety, and depression in breast cancer. Psychosomatics 59, 464–471. doi: 10.1016/j.psym.2018.01.005, PMID: 29525522 PMC6067989

[ref36] Mickey R. M. Greenland S. (1989). The impact of confounder selection criteria on effect estimation. Am. J. Epidemiol. 129, 125–137. doi: 10.1093/oxfordjournals.aje.a115101, PMID: 2910056

[ref37] Naser A. Y. Hameed A. N. Mustafa N. Alwafi H. Dahmash E. Z. Alyami H. S.. (2021). Depression and anxiety in patients with cancer: a cross-sectional study. Front. Psychol. 12:585534. doi: 10.3389/fpsyg.2021.585534, PMID: 33935849 PMC8081978

[ref38] Nasser Ibrahim Abu El N. MysoonKhalil Abu El N. (2015). Quality of life in prostate cancer survivors in developing countries: the case of the Gaza Strip, Palestine. Nursing Practice Today, 1. Available at: https://npt.tums.ac.ir/index.php/npt/article/view/9 (Accessed March 15, 2022).

[ref39] O’Connor M. Drummond F. O’Donovan B. Donnelly C. (2019). The unmet needs of cancer survivors in Ireland: A scoping review 2019. Available at: https://www.ncri.ie/sites/ncri/files/pubs/HSE%20Report%203%20-%20Unmet%20needs%20of%20cancer%20survivors%20in%20Ireland%20Final%20Version.pdf (Accessed April 24, 2022).

[ref40] Omran S. Khader Y. McMillan S. (2017). Symptom clusters and quality of life in hospice patients with cancer. Asian Pac. J. Cancer Prev. 18, 2387–2393. doi: 10.22034/APJCP.2017.18.9.2387, PMID: 28950683 PMC5720641

[ref41] Ownby K. K. (2019). Use of the distress thermometer in clinical practice. J. Adv. Pract. Oncol. 10, 175–179. doi: 10.6004/jadpro.2019.10.2.7 31538028 PMC6750919

[ref42] Palestinian Center for Human Rights (2017). 2 million Palestinians denied traveling and movement due to closure of Gaza border crossing. Availavle at: https://pchrgaza.org/en/2-million-palestinians-denied-traveling-and-movement-due-to-closure-of-gaza-border-crossing/ [Accessed January 27, 2017].

[ref43] Palestinian Ministry of Health (2022). Annual report, 2021. Availavle at: https://rb.gy/ra5tf7 [Accessed March 15, 2017].

[ref44] Renovanz M. Gutenberg A. Haug M. Strittmatter E. Mazur J. Nadji-Ohl M.. (2013). Postsurgical screening for psychosocial disorders in neurooncological patients. Acta Neurochir. 155, 2255–2261. doi: 10.1007/s00701-013-1884-9, PMID: 24078064

[ref45] Ryan D. A. Gallagher P. Wright S. al., e. (2012). Sensitivity and specificity of the distress thermometer and a two-item depression screen (patient health Questionnaire-2) with a ‘help' question for psychological distress and psychiatric morbidity in patients with advanced cancer. Psycho-Oncology 21, 1275–1284. doi: 10.1002/pon.2042, PMID: 21919118

[ref46] Sabateen A. Khalil M. Abu El Hawa M. Peeperkorn R. Mataria A. Ravaghi H. (2022). Proactive innovation in a prolonged conflict setting: facing covid-19 in a specialized cancer hospital in Palestine. Front. Public Health 10:873219. doi: 10.3389/fpubh.2022.873219, PMID: 35433608 PMC9010458

[ref47] Salah M. Reyala M. A. Al Jerjawy M. (2018). Quality of life among children with cancer in Gaza strip. Am. J. Health Res. 6, 119–125. doi: 10.11648/j.ajhr.20180605.12

[ref48] Shamallakh A. N. Imam A. M. (2017). Quality of life in patients with cancer in the Gaza strip: a cross-sectional study. Lancet 390:S21. doi: 10.1016/S0140-6736(17)32072-X

[ref49] Slavich G. M. Irwin M. R. (2014). From stress to inflammation and major depressive disorder: a social signal transduction theory of depression. Psychol. Bull. 140, 774–815. doi: 10.1037/a0035302, PMID: 24417575 PMC4006295

[ref50] Steel Z. Chey T. Silove D. Marnane C. Bryant R. A. van Ommeren M. (2009). Association of torture and other potentially traumatic events with mental health outcomes among populations exposed to mass conflict and displacement: a systematic review and meta-analysis. JAMA 302, 537–549. doi: 10.1001/jama.2009.1132, PMID: 19654388

[ref51] Terkawi A. S. Tsang S. AlKahtani G. J. Al-Mousa S. H. Al Musaed S. AlZoraigi U. S.. (2017). Development and validation of Arabic version of the hospital anxiety and depression scale. Saudi J Anaesth 11, 11–s18. doi: 10.4103/sja.SJA_43_17, PMID: 28616000 PMC5463562

[ref52] Thompson S. (2012). Sampling. *3rd Edn*. 2012 John Wiley & Sons, Inc. John Wiley & Sons, Inc. 472.

[ref53] van Laarhoven H. W. Schilderman J. Bleijenberg G. Donders R. Vissers K. C. Verhagen C. A.. (2011). Coping, quality of life, depression, and hopelessness in cancer patients in a curative and palliative, end-of-life care setting. Cancer Nurs. 34, 302–314. doi: 10.1097/NCC.0b013e3181f9a040, PMID: 21116179

[ref54] Veeraiah S. Kayser K. Sudhakar R. (2022). Psychosocial factors influencing distress among cancer patients in South India. J. Psychosoc. Oncol. Res. Pract. 4:e067. doi: 10.1097/OR9.0000000000000067

[ref55] World Health Organisation (2020). Health access barriers for patients in the occupied Palestinian territory. Available at: http://www.emro.who.int/images/stories/palestine/documents/nov_2019_monthly.pdf?ua=1&ua=1 [Accessed April 22, 2020].

[ref56] World Health Organization. (2019). Gaza patients’ painful journey to cancer treatment. Available at: http://www.emro.who.int/pse/palestine-news/gaza-patients-painful-journey-to-cancer-treatment.html [Accessed April 12, 2020].

[ref57] Yee M. K. Sereika S. M. Bender C. M. Brufsky A. M. Connolly M. C. Rosenzweig M. Q. (2017). Symptom incidence, distress, cancer-related distress, and adherence to chemotherapy among African American women with breast cancer. Cancer 123, 2061–2069. doi: 10.1002/cncr.30575, PMID: 28199006

[ref58] Zigmond A. S. Snaith R. P. (1983). The hospital anxiety and depression scale. Acta Psychiatr. Scand. 67, 361–370. doi: 10.1111/j.1600-0447.1983.tb09716.x 6880820

